# Competition for zinc binding in the host-pathogen interaction

**DOI:** 10.3389/fcimb.2013.00108

**Published:** 2013-12-24

**Authors:** Mauro Cerasi, Serena Ammendola, Andrea Battistoni

**Affiliations:** ^1^Dipartimento di Biologia, Università di Roma Tor VergataRome, Italy; ^2^Istituto Nazionale Biostrutture e Biosistemi, Consorzio InteruniversitarioRome, Italy

**Keywords:** zinc uptake, ZnuABC, antibacterial therapies, metal cofactor, host-pathogen interaction, *Salmonella enterica*, zinc transporter, nutritional immunity

## Abstract

Due to its favorable chemical properties, zinc is used as a structural or catalytic cofactor in a very large number of proteins. Despite the apparent abundance of this metal in all cell types, the intracellular pool of loosely bound zinc ions available for biological exchanges is in the picomolar range and nearly all zinc is tightly bound to proteins. In addition, to limit bacterial growth, some zinc-sequestering proteins are produced by eukaryotic hosts in response to infections. Therefore, to grow and multiply in the infected host, bacterial pathogens must produce high affinity zinc importers, such as the ZnuABC transporter which is present in most Gram-negative bacteria. Studies carried in different bacterial species have established that disruption of ZnuABC is usually associated with a remarkable loss of pathogenicity. The critical involvement of zinc in a plethora of metabolic and virulence pathways and the presence of very low number of zinc importers in most bacterial species mark zinc homeostasis as a very promising target for the development of novel antimicrobial strategies.

## Zinc: chemical properties and role in bacterial proteins

Among transition metals, zinc is likely the one which is used as a structural or catalytic cofactor in the wider number of proteins. The widespread use of zinc in proteins can be related to its peculiar chemical properties (Andreini et al., 2008). Unlike the other biological relevant transition metals (Fe^2+^, Mn^2+^, Cu^2+^, Ni^2+^) the zinc ion (Zn^2+^) has a filled *d* orbital and, therefore, it is redox stable. Zinc mainly participates to catalytic reactions by acting as a Lewis acid able to accept electron pairs or, as an alternative, by attracting or stabilizing negative charges of the substrates. Moreover, zinc binding to proteins is facilitated by its capability to form stable chemical bonds with nitrogen, oxygen and sulfur atoms and assume different coordination numbers. As a consequence zinc can be found in a large variety of distinct chemical environments, which may significantly modulate its reactivity. However, a potential problem of zinc is that it binds to proteins stronger than the other divalent metals (Irving and Williams, 1948) and, therefore, cells maintain the intracellular pool of “free” metal at very low levels to prevent its unspecific binding to proteins (Colvin et al., 2010).

Different studies have attempted to measure the amount of zinc in bacteria. It has been shown that microorganisms have a remarkable capability to modify their intracellular zinc content in response to variations in the environmental availability of the metal (Outten and O'Halloran, 2001; Garmory and Titball, 2004) and that the total cellular zinc in bacteria growing in rich media is in the submillimolar range (10^−4^ M), i.e., a concentration comparable to that usually observed in most eukaryotic cells (Eide, 2006). More complex is to obtain a careful evaluation of the intracellular pool of metal ions not tightly bound to proteins. *In vitro* studies carried out with purified zinc-responding transcriptional regulators have initially suggested that cellular “free” zinc levels are in the femtomolar range, i.e., around 10^−15^ M (Outten and O'Halloran, 2001). However, recent studies involving protein-based ratiometric biosensors have established that *in vivo* the concentration of intracellular exchangeable zinc is around 20 pM, i.e., 2 × 10^−11^M (Wang et al., 2011). Picomolar values of “free” zinc have been reported also in several eukaryotic systems (Colvin et al., 2010).

Interestingly, although the zinc concentration in bacterial cells is close to that of iron, a significant fraction of iron may be found in association to proteins such as ferritins, bacterioferritins or DPS (Andrews et al., 2003), whereas *bona fide* zinc-storage proteins are present only in a few bacteria (Blindauer et al., 2002). An experimental attempt to explore the complexity of the bacterial zinc proteome has shown that more than 3% of the proteins expressed in *Escherichia coli* contain zinc (Katayama et al., 2002), whereas bioinformatics investigations have revealed that about 5% of all bacterial proteins contain recognizable zinc-binding sites (Andreini et al., 2006). This means that an *E. coli* cell with about 4300 protein-encoding genes contains more than 200 zinc-binding proteins. These figures, however, are not sufficient to have an accurate idea of the actual importance of this metal in the physiology of a bacterial cell. In fact, in addition to being an essential cofactor in a large number of enzymes involved in central metabolic pathways, zinc is bound to several proteins involved in the management of gene expression, including some ribosomal proteins (Hensley et al., 2011), RNA polymerases (Scrutton et al., 1971), tRNA synthetases (Miller et al., 1991), sigma factor interacting proteins (Campbell et al., 2007) and zinc responding transcriptional factors (Chivers, 2007). Moreover, zinc is involved in other crucial processes, including DNA repair (Kropachev et al., 2006), response to oxidative stress (Ortiz De Orue Lucana et al., 2012), antibiotic resistance (Meini et al., 2013) and production of virulence-related proteins (Ammendola et al., 2008). It follows that changes in the intracellular concentrations of zinc can have pleiotropic effects on the composition of the bacterial proteome, involving changes in the expression and activity of zinc-containing proteins as well as of proteins which do not employ this cofactor.

## Bacterial zinc uptake systems and response to zinc shortage

**Figure 1:**
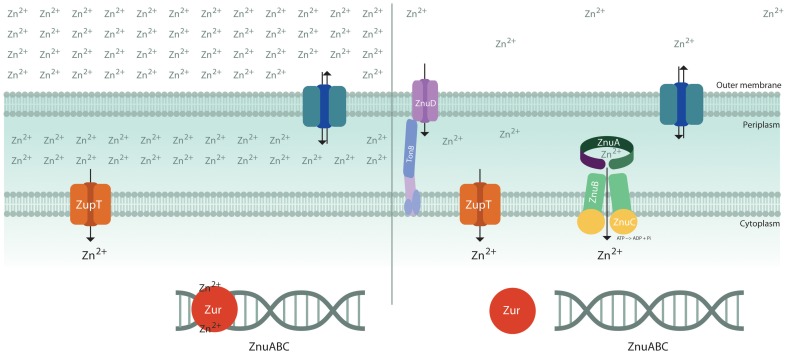
**Schematic diagram of transporters involved in zinc uptake.** The bacterial outer membrane is thought to be permeable to hydrophilic solutes of <600 dalton (Nikaido and Vaara, 1985) and, therefore, zinc concentration in the periplasmic space is largely dependent on zinc availability in the environment. Under zinc replete conditions (left), the metal is imported through low affinity import systems, such as ZupT, and Zur inhibits the expression of the importer ZnuABC. Under conditions of zinc shortage (right), apoZur is unable to bind DNA and the high affinity zinc importer ZnuABC is expressed. *Neisseria meningitidis* expresses a Zur-regulated TonB-dependent outer membrane protein, ZnuD, involved in zinc uptake.

Although in bacteria exposed to high levels of zinc the metal may enter through a large number of unspecific channels, only a few metal transporters are known to mediate the specific uptake of zinc (Hantke, 2005) (Figure 1). Some recent studies on the pneumococcal PsaA protein involved in manganese uptake have provided interesting hints to understand the mechanisms ensuring specificity in transition metal import (McDevitt et al., 2011; Counago et al., 2013). PsaA may bind either manganese or other first-row transition metals, but, due to the propensity of zinc to form stable complexes with proteins (Irving and Williams, 1948) it competitively affects Mn^2+^ binding and locks the protein in a conformation which prevents the entry either of zinc or of manganese. This observation provides an explanation for the ability of zinc to inhibit pneumococcal growth (McDevitt et al., 2011) and an elegant example of the strategies used by living cells to guarantee the correct uptake of specific metal ions (Waldron and Robinson, 2009).

In several Gram-negative bacteria growing in metal replete conditions, zinc uptake is thought to be primarily mediated by ZupT, a constitutively expressed low affinity transporter belonging to the ZIP (ZRT-, IRT-like Protein) protein family (Grass et al., 2002). This metal permease has a broad metal specificity, but it displays a clear preference for zinc over other divalent metals (Grass et al., 2005). ZupT depends on the proton motive force to energize zinc import (Karlinsey et al., 2010; Taudte and Grass, 2010).

The response to zinc paucity is controlled through the coordinated expression of a set of genes regulated by the transcriptional factor Zur, which may bind two or more zinc ions, depending on the species (Outten et al., 2001; Lucarelli et al., 2007; Shin et al., 2011). One atom of zinc serves a structural role, whereas the other atom(s) favors the folding of a DNA-binding domain enabling the protein to tightly bind to a consensus sequence located in the promoter of said genes. In contrast, when the intracellular zinc content decreases, zinc-devoid Zur is no longer able to stably interact with DNA and to repress transcription. The number of known Zur–regulated genes changes in different bacteria, but in all species they include a small operon encoding for the components of ZnuABC, a high affinity zinc importer of the ABC family, and one or more genes encoding for paralogs of zinc-containing ribosomal proteins (Panina, 2003; Graham et al., 2009; Li et al., 2009; Lim et al., 2013). The ZnuABC uptake system is composed of three proteins: the ZnuB channel, the ZnuC ATPase component which provides the energy necessary for ion transport through the inner membrane, and ZnuA, a soluble protein which captures Zn(II) in the periplasm with high efficiency and delivers it to ZnuB (Patzer and Hantke, 1998). In some bacteria there is also an accessory component of the ZnuABC transporter, ZinT, which is known to form a complex with ZnuA in presence of zinc and is thought to enhance ZnuA ability to recruit zinc (Petrarca et al., 2010; Ilari et al., 2013). A similar zinc uptake system (AdcABC) can be found in pneumococci and some other Gram-positive bacteria, where the lipoprotein AdcA is characterized by two structural domains that show clear sequence and structural homology with ZnuA and ZinT, respectively (Dintilhac et al., 1997; Panina et al., 2003).

An interesting facet of the Zur-mediated response to zinc shortage is the substitution of ribosomal proteins containing zinc with homologous proteins lacking the zinc-binding motif. This change in ribosomal structure reduces the metal requirements of bacterial cells, as the majority of intracellular zinc is thought to be associated to ribosomes (Hensley et al., 2011). Moreover, the production of zinc-independent ribosomal proteins may be useful to mobilize a relevant amount of metal from pre-existing ribosomes and facilitates the adaptation to zinc-limiting conditions (Gabriel and Helmann, 2009). From this point of view, the ribosome may be described as a zinc storage protein complex. Additional paralogs of zinc-containing proteins have been identified in several bacteria (Haas et al., 2009) and include a homolog of the transcriptional factor DksA which is involved in the control of the bacterial response to stress and starvation (Blaby-Haas et al., 2011).

The outer membrane of Gram-negative bacteria allows the passive diffusion of low molecular weight molecules. However, a mechanism of nutrient uptake solely based on diffusion may be hardly able to ensure the adequate absorption of elements which are poorly available in the environment. In recent years, an outer membrane TonB-dependent receptor involved in zinc uptake has been identified in *Neisseria meningitidis* and some other Gram-negative bacteria (Stork et al., 2010). This protein, denominated ZnuD, mediates either zinc or heme uptake and is regulated either by Zur or by Fur (Kumar et al., 2012; Pawlik et al., 2012). The pneumococcal surface protein PhtD has been proposed to play a functionally similar role in favoring zinc uptake through AdcAII (Loisel et al., 2011). No outer membrane zinc receptors have been so far identified in Enterobacteria or in other Gram-negative bacteria. However, it has been observed that apo-ZinT can be extruded outside the cell, suggesting that it could have some role in the acquisition of zinc from the environment (Ho et al., 2008; Gabbianelli et al., 2011).

It should also be noted that a few bacterial species have been shown to express more than one high affinity zinc uptake systems. This is the case of *Listeria* *monocytogenes* which expresses two ABC-type zinc importers (ZnuABC and ZurAM), both contributing to full virulence (Corbett et al., 2012) and of non-typeable *Haemophilus influenzae*, where the zinc binding system ZevAB facilitates growth in zinc-limiting conditions and lung colonization in infected mice (Rosadini et al., 2011). Similarly, disruption of *znuA* in *Pseudomonas aeruginosa* results in a very limited growth defect under zinc-limiting conditions (Ellison et al., 2013), possibly due to the expression of a zinc-importing P-type ATPase (Lewinson et al., 2009).

## Zinc in the host-pathogen interaction

Whereas the competition for iron acquisition has been recognized as a key element of the host pathogen interaction for a long time, only in recent years the efficient uptake of other divalent metals has emerged to play a comparable role (Kehl-Fie and Skaar, 2010). In particular the importance of zinc has become clear through a series of investigations which have established that deletion of the *znuABC* genes not only decrease bacterial ability to growth in *in vitro* environments poor of this metal, but also dramatically affects their pathogenicity. Bacterial pathogens which have been shown to critically depend on ZnuABC to infect their hosts include *Acinetobacter baumannii* (Hood et al., 2012), *Brucella abortus* (Kim et al., 2004; Yang et al., 2006), *Campylobacter jejuni* (Davis et al., 2009), pathogenic *E. coli* strains (Sabri et al., 2009; Gabbianelli et al., 2011), *H. ducreyi* (Lewis et al., 1999), *Moraxella catarrhalis* (Murphy et al., 2013), *Pasteurella multocida* (Garrido et al., 2003), *Salmonella enterica* (Campoy et al., 2002; Ammendola et al., 2007) and *Yersinia ruckeri* (Dahiya and Stevenson, 2010). In contrast, while being required for zinc uptake *in vitro*, ZnuABC does not contribute to *Y. pestis* virulence (Desrosiers et al., 2010) and provides only a limited advantage to *Proteus mirabilis* during urinary tract infections (Nielubowicz et al., 2010). It is not yet clear whether these bacteria possess additional zinc importers or if they show limited zinc requirements during infections.

Probably, the previous underestimation of the importance of zinc in the interaction between bacteria and their hosts can be largely attributed to the apparent abundance of this element in all tissues. In fact, high levels of zinc are present either within cells or in the plasma, where most of the metal is loosely associated to proteins (Zalewski et al., 2006) and, therefore, potentially available for invading microorganisms. However, it should be noted that a typical feature of the early response to the infection is the rapid fall of plasma zinc concentration, accompanied by zinc accumulation in the liver. Redistribution of zinc among the various tissues is regulated by a lipopolysaccharide-induced cytokines cascade (with IL-6 playing a central role) which stimulates increased synthesis of acute phase proteins, such as metallothionein, and the hepatic uptake of the metal through the induction of the solute carrier 39 (SLC39) protein ZIP14 (Liuzzi, 2005). In view of the importance of the ZnuABC transporter in bacterial zinc uptake during infections, this feature of the acute phase response appears as an adaptive mechanism intended to deprive pathogens of zinc.

The role of the ZnuABC transporter has been investigated in details in *S. enterica* serovar Typhimurium (*S.* Typhimurium). Expression of *znuABC* is repressed in *S.* Typhimurium cultivated in synthetic media containing zinc concentrations as low as 1 μM. In contrast, the *znuABC* operon is strongly induced in bacteria recovered from the spleens of infected mice or from cultured epithelial or macrophagic cells (Ammendola et al., 2007). These observations suggest that zinc bound to proteins is not easily available for invading bacteria and that ZnuABC is required to have rapid access to the pool of “free” zinc. More recently, it has been shown that during gut infections ZnuABC confers resistance to the antimicrobial protein calprotectin (Liu et al., 2012). Calprotectin is a neutrophilic protein of the S100 family of calcium binding proteins, that is abundantly released at sites of infection to control the multiplication of pathogens by the sequestration of zinc and manganese (Kehl-Fie and Skaar, 2010). In support to the experimental evidence that calprotectin starves bacteria for metal ions, structural studies have confirmed that calprotectin possesses two distinct binding sites for transition metals, one of which is specific for zinc and the other one may accommodate either zinc or manganese (Brunjes Brophy et al., 2013; Damo et al., 2013). Bacteria expressing ZnuABC are able to resist to such antimicrobial strategy and this favor their growth over competing microbes in the inflamed gut (Gielda and Dirita, 2012; Liu et al., 2012). Taken together, these studies suggest that zinc acquisition through the ZnuABC transporter is essential for the colonization of *Salmonella* in mice, and provide a parallelism between the mechanisms of iron and zinc sequestration in the host-pathogen relationships. It is worth noting that calprotectin is not the unique S100 protein involved in zinc sequestration. In fact, a comparable function has been proposed for the antibacterial protein psoriasin (S100A7), which protects human skin from *E. coli* infections (Glaser et al., 2005).

Although, the above mentioned studies have suggested that zinc sequestration is a strategy widely used by vertebrates to control microbial infections, a few recent observations have revealed an alternative way to use zinc in host defense. In fact, it has been shown that human macrophages control mycobacteria by elevating zinc levels in the bacteria-containing phagosomes (Botella et al., 2011). This process is dependent on reactive oxygen species generated by the phagocytic NADPH oxidase and involves the mobilization of zinc from intracellular stores. Mycobacterial resistance to zinc intoxication in macrophages relies on their ability to induce the expression of heavy metal efflux P-type ATPases, which prevent the intracellular accumulation of zinc at toxic levels. Structurally homologous zinc efflux pumps have been identified in a large number of bacteria, including *E. coli*, where the P-type zinc exporter ZntA has been proved to be critical for zinc tolerance (Beard et al., 1997; Rensing et al., 1997) and for the maintaining of appropriate levels of intracellular zinc (Wang et al., 2012). Whereas mycobacteria lacking the efflux pump CtpC or *E. coli* cells devoid of ZntA display a reduced ability to survive in human macrophages, disruption of CtpC does not affect the ability of *M. tuberculosis* to infect mice (Botella et al., 2011).

To add to the complexity, a mobilization of zinc in the opposite direction to that found in response to mycobacteria has been observed in murine macrophages infected with the fungus *Histoplasma capsulatum* (Subramanian Vignesh et al., 2013). In this case, phagosomes are deprived of zinc and the metal accumulates in the Golgi or in the cytoplasm, in association to metallothioneins. Further studies are needed to understand whether these different responses depend on the cross-talk between each specific microorganism and phagocytic cells, or whether the ability to poison bacteria through an excess of zinc is a prerogative of human macrophages.

## Zinc homeostasis as a target for antibacterial therapies

Different studies have proposed that ABC transporters could be effective targets for the development of novel antibacterial drugs or vaccines (Garmory and Titball, 2004; Counago et al., 2012). In this view, ZnuABC appears as a particularly promising candidate.

Whereas a large number of pathogens produce multiple metal import systems that enable the uptake of different iron forms (reduced or oxidized, “free” or bound to proteins or to heme), in most bacteria there is only one high affinity zinc importer. Moreover, it has been shown that *Salmonella* strains lacking the whole *znuABC* operon display the same dramatic loss of virulence of strains producing ZnuB and ZnuC, but lacking ZnuA (Petrarca et al., 2010). This finding indicates that the ability of ZnuA to effectively compete for zinc binding with other periplasmic proteins is critical to ensure zinc import in the cytoplasm (Berducci et al., 2004) and suggests that drugs targeting the soluble component of the transporter could block zinc import. This is a very interesting possibility because ZnuA is a suitable substrate for biochemical and structural studies (Banerjee et al., 2003; Chandra et al., 2007; Wei et al., 2007; Yatsunyk et al., 2008; Ilari et al., 2011; Castelli et al., 2013). It is worth noting that several molecules able to interfere with zinc uptake in *Candida albicans* have been recently identified through the screening of a small-molecule library of 2000 compounds (Simm et al., 2011), thus providing a proof of concept that it is possible to pharmacologically target zinc homeostasis in pathogenic microorganisms.

Bacterial mutant strains unable to produce the ZnuABC transporter are also putative candidate for the development of live–attenuated vaccines. It has been shown that a *S*. Typhimurium *znuABC* strain is able to induce short lasting infections in mice which induce a solid and durable immune-based protection against virulent strains of *S.* Typhimurium (Pasquali et al., 2008; Pesciaroli et al., 2011). The same strain proved to be attenuated, immunogenic and protective also in pigs (Gradassi et al., 2013; Pesciaroli et al., 2013). Similarly, it has been shown that vaccination with *Brucella* *znuA* mutants protects mice from *Brucella* infections (Yang et al., 2006; Clapp et al., 2011). In addition, it has been shown that ZnuD is able to elicit the production of antibodies triggering complement-mediated killing of several *Neisseria meningitidis* serogroup B strains, suggesting that it is a promising candidate for the generation of an effective vaccine (Hubert et al., 2013). Taken together, these studies suggest that zinc homeostasis offers interesting options to generate vaccines against different pathogenic bacteria.

### Conflict of interest statement

The authors declare that the research was conducted in the absence of any commercial or financial relationships that could be construed as a potential conflict of interest.
